# Biopsy of undescended testis mimicking a metastatic inguinal lymph node – Ouch, it hurts! Lesion: A case report

**DOI:** 10.1097/MD.0000000000043526

**Published:** 2025-08-08

**Authors:** Young Kwang Lee, Jae Seok Jeong, Yeon-Hee Han, Ho Sung Park, Yong Chul Lee, Eun Hae Park

**Affiliations:** a Department of Radiology, Jeonbuk National University Medical School, Jeonju, Republic of Korea; b Department of Radiology, OK Cham Hospital, Guri-si, Gyeonggi-do, Republic of Korea; c Research Institute of Clinical Medicine of Jeonbuk National University-Biomedical Research Institute of Jeonbuk National University Hospital, Jeonju, Republic of Korea; d Department of Internal Medicine and Research Center for Pulmonary Disorders, Jeonbuk National University Medical School, Jeonju, Republic of Korea; e Department of Nuclear Medicine, Jeonbuk National University Hospital, Jeonju, Republic of Korea; f Department of Pathology, Jeonbuk National University Medical School, Jeonju, Republic of Korea.

**Keywords:** case report, cryptorchidism, metastasis, ultrasound

## Abstract

**Rationale:**

Undescended testis (cryptorchidism) is the most common congenital anomaly of the urogenital system. Testicular tissue exhibits variable fluorodeoxyglucose (FDG) uptake and may demonstrate a physiologically increased standardized uptake value. In cases of undescended testis with FDG uptake, immediate recognition can be challenging, increasing the risk of misinterpretation as a pathological lymph node.

**Patient concerns:**

A 45-year-old male inpatient, was referred for ultrasound-guided biopsy of a right inguinal structure suspected to be a metastatic lymph node. Three weeks earlier, high-resolution computed tomography incidentally detected a cavitary pulmonary nodule, which was later confirmed as an adenocarcinoma through biopsy. For staging, an F-18 FDG positron emission tomography–computed tomography identified a solitary hypermetabolic inguinal lesion, raising suspicion of metastasis.

**Diagnosis:**

Histopathological examination confirmed that the structure was an undescended testis, rather than a metastatic lymph node.

**Interventions:**

The patient underwent an ultrasound-guided core needle biopsy under local anesthesia.

**Outcomes:**

The patient underwent a left upper lobectomy and had shown no signs of recurrence or bone metastasis to date.

**Lessons:**

This case highlights the potential for misdiagnosis of an undescended testis as an inguinal metastatic lymph node due to physiological FDG uptake. Awareness of key imaging and clinical features, such as testicular mobility, relative isoechogenicity, and pain during biopsy, can help prevent unnecessary procedures and ensure an accurate diagnosis.

## 1. Introduction

Undescended testis are the most common urogenital system disorder. The testis is known to have variable fluorodeoxyglucose (FDG) uptake and often shows physiologically increased standardized uptake value (SUV). In cases of undescended testis with FDG uptake, it may be difficult to detect immediately, and there is a potential for misinterpretation as a pathologic lymph node. Here, we report a case of unilateral undescended testis that mimicked a metastatic inguinal lymph node due to moderate FDG uptake (SUV_max_ = 4.10) on an F-18 FDG positron emission tomography–computed tomography (PET/CT) scan performed as part of a recent lung cancer workup. A biopsy of the structure was performed.

## 2. Case description

**Figure 1.**
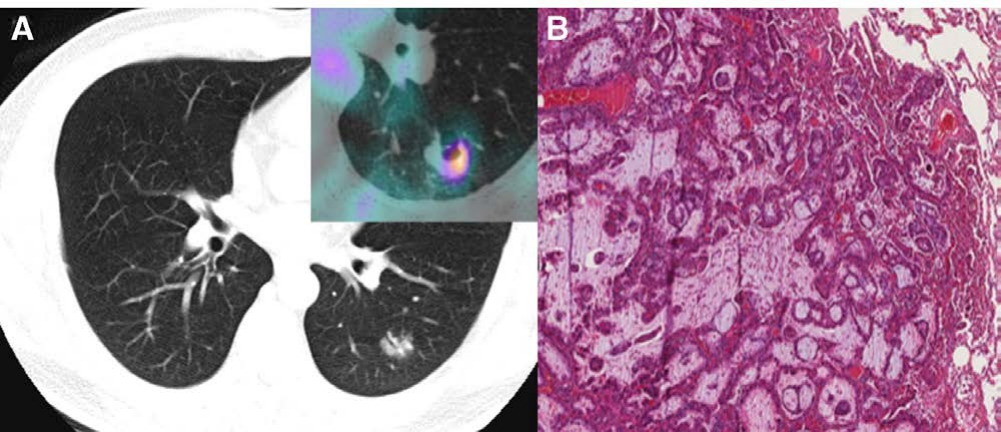
(A) Chest CT reveals a 1.9 × 1.5 cm lobulated cavitary lesion with well-defined margins and an air bronchogram in the superior segment of the left lower lobe. In the upper right corner, 2-deoxy-2-[18F]fluoro-D-glucose (FDG) positron emission tomography (PET)/CT shows hypermetabolism in the cavitary pulmonary nodule, with an SUV_max_ of 3.29. (B) Histologic examination of the pulmonary mass, viewed at low magnification, reveals a relatively well-circumscribed lung tumor with mucin production (H&E stain, ×40), confirming the diagnosis of pulmonary adenocarcinoma. CT = positron emission tomography–computed tomography, SUV = standardized uptake value.

A 45-year-old male patient was referred to the radiology department for ultrasound-guided biopsy of a right inguinal structure suspected to be a metastatic lymph node. Approximately 3 weeks earlier, high-resolution CT incidentally detected a solitary pulmonary nodule: a 2 cm cavitary lesion in the superior segment of the left lower lobe (Fig. 1A). Enlarged mediastinal lymph nodes were not observed.

The patient was hospitalized for further evaluation, and fluoroscopy-guided percutaneous biopsy of the pulmonary nodule was performed. Histopathological analysis revealed glandular structures composed of mucin-producing tumor cells, confirming the diagnosis of adenocarcinoma (Fig. 1B).

**Figure 2.**
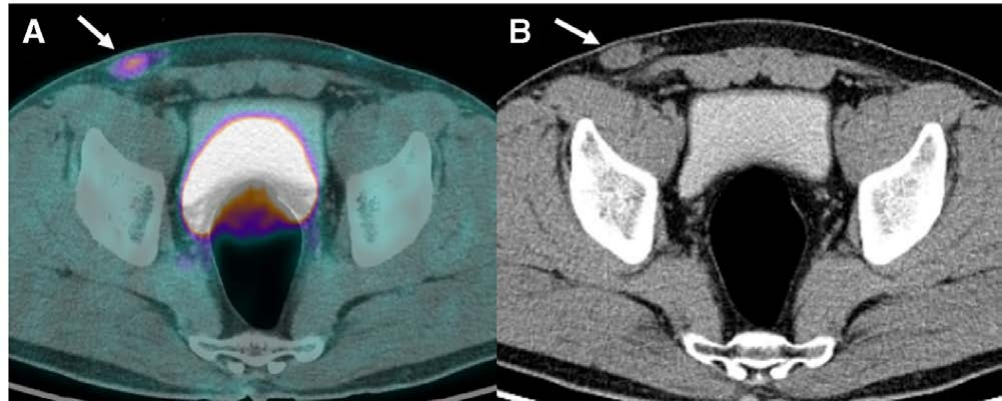
(A and B) FDG PET/CT demonstrates an oval nodular structure in the right inguinal area (arrows) with moderate FDG uptake (SUV_max_ = 4.10). FDG = fluorodeoxyglucose, PET/CT = positron emission tomography–computed tomography, SUV = standardized uptake value.

For staging, F-18 FDG PET/CT was performed, revealing moderate FDG uptake (SUV_max_ = 4.10) in a 2.5 cm oval structure in the right inguinal region, which was reported as a suspicious metastatic lymph node (Fig. 2A and B). This was the only suspected metastatic lesion, and no other abnormal FDG uptake, suggestive of distant metastasis, was identified.

**Figure 3.**
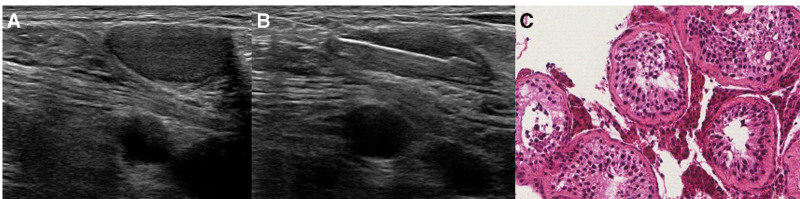
(A) Ultrasound of the right inguinal mass reveals a well-defined, isoechoic oval structure with obliteration of the hyperechoic fatty hilum and cortical thickening, raising suspicion for a metastatic lymph node. (B) The biopsy needle is positioned at the center of the structure. (C) Histological examination of the specimen revealed seminiferous tubules with a reduced number of spermatocytes and spermatids, along with marked thickening of the tunica propria, confirming the diagnosis of cryptorchid testis (H&E staining, ×200).

An experienced musculoskeletal radiologist performed ultrasound-guided biopsy of the right inguinal structure. On ultrasound, a well-defined oval structure, measuring approximately 2.8 × 0.9 × 1.0 cm, was observed in the right inguinal region, in close proximity to the neurovascular bundle. A hyperechoic fatty hilum was absent, suggesting inguinal lymph node metastasis (Fig. 3A). The structure showed focal peripheral vascularity.

However, during ultrasound-guided biopsy (Fig. 3B), unusual findings were observed compared with those of typical inguinal metastatic lymph node biopsies. The structure was more mobile than expected. In response, the assistant blocked the needle’s opposite trajectory with 2 fingers. Additionally, the patient experienced severe pain despite administration of sufficient local anesthesia.

This unexpected finding, which surprised both the radiologist and oncologist, was confirmed by pathological examination. Histological examination revealed a tubular structure with seminiferous tubules containing a reduced number of spermatocytes and spermatids, along with marked thickening of the tunica propria, confirming the inguinal structure as testicular tissue and diagnosing cryptorchidism (Fig. 3C).

A retrospective review of F-18 FDG PET/CT revealed an empty scrotum, which had been overlooked by nuclear medicine physician, oncologist, and radiologist. Following histopathological confirmation, the patient reported lifelong awareness of right-sided scrotal emptiness. However, this detail had not been disclosed during the initial consultation and was consequently absent from the medical records. The patient reported no additional pain following the procedure, and no complications including hematoma or infection were observed.

## 3. Discussion

In the present case, unnecessary biopsy was performed on an overlooked undescended testis with moderate FDG uptake, mimicking a metastatic inguinal lymph node. Cryptorchidism is the most common disorder of the urogenital system.^[1–3]^ To our knowledge, this is the first reported case of a biopsy performed on an undescended testis that mimicked a metastatic inguinal lymph node on PET/CT. Reported complications of biopsy of an undescended testis, including hemorrhage and infection, are rare.^[4]^ Although an unnecessary procedure was performed, the patient experienced unexpected benefits. Testicular cancer has a higher incidence of cryptorchidism,^[5]^ and laparoscopic-guided biopsy may be considered in suspicious cases. However, despite FDG uptake, pathological examination revealed normal testicular tissue without tumor cells.

The testis exhibits variable FDG uptake and often demonstrates a physiologically increased SUV. FDG uptake decreased with age; men aged 30 to 39 years had an average SUV of 2.81 ± 0.43, whereas men aged 80 to 89 years had a lower average SUV of 2.18 ± 0.45.^[6]^ Unlike normal testes, cryptorchid testes demonstrate greater variability in uptake, primarily due to prolonged exposure to elevated intra-abdominal or inguinal temperatures.^[7]^ These environmental conditions can impair spermatogenesis and induce histopathological changes such as tubular atrophy or fibrosis, resulting in altered glucose metabolism.^[8,9]^ Consequently, FDG uptake in undescended testes may appear asymmetrical, focal, or elevated (features that can closely mimic malignant or inflammatory lesions on imaging).^[10]^

Given this variability, undescended testes with FDG uptake can be difficult to recognize on PET/CT, especially when there is no known history of cryptorchidism. This diagnostic uncertainty increases the likelihood of misinterpreting the lesion as a pathological lymph node, as observed in the present case.^[11,12]^ Previous reports in which an undescended testis was mistakenly interpreted as a metastatic lymph node on PET/CT have identified various primary malignancies, including melanoma, colon cancer with liver metastasis, and lymphoma.^[11–14]^ The degree of FDG uptake in these cases varied from mild to moderate, with a maximum SUV of 3.1, as reported by Groheux et al,^[11]^ which was initially interpreted as indicative of residual lymphoma. However, the SUV_max_ in the present case was 4.10 (the highest reported to date in the English literature) which further complicated the initial interpretation.

Groheux et al identified several factors that may help prevent the misinterpretation of an undescended testis as a metastatic lymph node, including its continuity with the spermatic cord, the presence of only one testis in the scrotal sac, differing treatment responses compared to other foci, and skipped lesions.^[11]^ In the present study, we further highlight additional pitfalls in ultrasound interpretation. Awareness of these issues may help reduce the risk of misinterpretation in future studies.

The first consideration is echogenicity. A critical ultrasound feature distinguishing testicular tissue from lymph nodes is the presence of the mediastinum testis, which appears as a linear echogenic band running across the posteromedial aspect of the testis. This echogenic structure represents the invagination of the tunica albuginea forming an incomplete septum that contains blood vessels and ducts, and its identification is considered essential for accurate testicular diagnosis.^[15]^ In contrast, lymph nodes lack this specific echogenic linear structure and typically present as hypoechoic masses with or without a fatty hilum. Ultrasound findings of metastatic lymph nodes typically include distinct focal cortical bulging, diffuse or eccentric cortical thickening, round hypoechoic nodes, effacement of the fatty hilum, and complete or partial nodal replacement with a mass. The upper limit for the short-axis diameter of a reactive lymph node in adults is 10 to 20 mm.^[16]^ On longitudinal ultrasound, a normal testis appears as an ovoid structure surrounded by an echogenic tunica albuginea and a visible mediastinum testisrunning along the midline. An undescended testis is typically smaller and may appear isoechoic or hypoechoic relative to a normally positioned testis.^[17]^

In the present case, several factors contributed to the radiologist’s misinterpretation of the structure as metastatic lymph nodes. The size of the structure (2.8 cm), oval shape, and homogeneous echogenicity suggest diffuse cortical thickening with obliteration of the fatty hilum. Additionally, the absence of a mediastinal-like structure, even upon retrospective review, further raises suspicion. Identification of this echogenic midline structure could have served as a crucial clue, indicating that the mass was testicular rather than nodal. The proximity of the structure to the neurovascular bundle, an area rich in lymph nodes, also contributed to this misinterpretation. However, the relatively isoechoic echogenicity of the structure should have been considered during the preprocedural ultrasound evaluation, as metastatic lymph node involvement is less likely. Although quantitative evaluation of echogenicity is lacking and the description is relative, metastatic lymph nodes are typically hypoechoic. Additionally, the general echogenicity of the testis increases during puberty, with adult testicular tissue typically demonstrating low-to-medium echogenicity.^[12,17]^

The second consideration is mobility. The testes descend from the abdominal cavity to the scrotal sac, and an undescended testis may be located at any point along this pathway, including the abdominal cavity, internal inguinal ring, inguinal canal, and external inguinal ring.^[18]^ Because the testis was located in the external inguinal canal in the present case, it could move within the canal.

Additionally, the testis is enclosed in a capsule consisting of the visceral tunica vaginalis and tunica albuginea, which are muscular fasciae. This can induce spasms when the needle approaches, leading to patient discomfort and unusual findings during biopsy.^[19]^

Building on these diagnostic challenges, clinicians should consider the possibility of an undescended testis when an oncologic patient presents with a palpable inguinal lesion.^[20]^ A structured diagnostic approach may begin with physical examination and review of the patient’s urological history, followed by imaging study. Scrotal and inguinal ultrasound or magnetic resonance imaging are usually preferred for evaluating urogenital anomalies such as cryptorchidism.^[21]^ However, in cancer patients, abdomino-pelvic CT (whether already performed for staging or newly obtained) can also be valuable when carefully reviewed for findings in both the inguinal and scrotal regions. If imaging or clinical signs suggest a urogenital abnormality, further evaluation with targeted imaging or urologic consultation should take place before biopsy. This stepwise process can reduce diagnostic errors, avoid unnecessary procedures, and improve patient safety.

Following the proposed diagnostic approach, the final key consideration was the patient’s report of extreme pain. Following the proposed diagnostic approach, the final key consideration was the patient’s report of extreme pain during the procedure. The testis receives its primary sensory innervation via nerve fibers originating from the renal and aortic (intermesenteric) plexuses, which travel alongside the testicular artery to the gonad. This visceral innervation explains why testicular pain during biopsy is often intense, poorly localized, and may radiate to the lower abdomen or groin (even when local anesthesia is used).^[22]^ These anatomical and physiological features account for the severe discomfort observed in our patient and highlight the importance of considering testicular origin in cases of unexplained inguinal pain during biopsy.^[23]^

In conclusion, an undescended testis may be overlooked, and physiological FDG uptake on PET/CT can lead to misinterpretation as an inguinal metastatic lymph node. However, if the inguinal structure is mobile, exhibits relative isoechogenicity compared to a metastatic lymph node, and is associated with pain, an undescended testis should be considered as a possible diagnosis.

## Acknowledgments

The authors would like to thank the participants of this study for their cooperation and understanding, without whom this study would have never been possible.

## Author contributions

**Conceptualization:** Jae Seok Jeong, Yong Chul Lee, Eun Hae Park.

**Data curation:** Young Kwang Lee, Yeon-Hee Han, Ho Sung Park, Eun Hae Park.

**Formal analysis:** Young Kwang Lee, Eun Hae Park.

**Funding acquisition:** Eun Hae Park.

**Investigation:** Jae Seok Jeong, Ho Sung Park.

**Methodology:** Jae Seok Jeong, Yong Chul Lee, Eun Hae Park.

**Software:** Yeon-Hee Han, Ho Sung Park, Eun Hae Park.

**Validation:** Jae Seok Jeong, Young Kwang Lee.

**Writing – original draft:** Jae Seok Jeong, Young Kwang Lee, Yeon-Hee Han, Eun Hae Park.

**Writing – review & editing:** Jae Seok Jeong, Young Kwang Lee, Yong Chul Lee, Eun Hae Park.
